# Stent Undersizing and Malapposition: Mechanical Causes of Acute and Late Stent Failure

**DOI:** 10.31083/RCM52926

**Published:** 2026-08-11

**Authors:** Fotios Toulgaridis, Nikolaos Tsiamis, Dimitrios Afendoulis, Flora Tsakirian, Sotirios Kotoulas, Christo Kole, Ioannis Andreou

**Affiliations:** ^1^Catheterization Laboratory, General Hospital of Attica “Sismanogleio”, 151 26 Athens, Greece; ^2^Unit of Structural and Valvular Heart Diseases, First Department of Cardiology, National and Kapodistrian University of Athens, General Hospital of Athens “Hippokration”, 115 27 Athens, Greece; ^3^Department of Cardiology, Asklepeion General Hospital, 166 73 Athens, Greece

**Keywords:** drug-eluting stents, coronary restenosis, prosthesis failure, percutaneous coronary intervention, tomography, optical coherence

## Abstract

Stent undersizing and malapposition are critical mechanical failures in contemporary percutaneous coronary intervention (PCI). These deficiencies create a substrate for both immediate and delayed complications, including acute stent thrombosis, delayed healing, and late stent restenosis. Despite technological advances, angiography often fails to detect clinically relevant malapposition, leading to a systematic underestimation of its prevalence and clinical impact. This review evaluates the mechanisms underlying stent undersizing and malapposition, the associated pathophysiological consequences, and the pivotal role of intracoronary imaging (ICI) in optimizing stent deployment and improving clinical outcomes. Key definitions include stent underexpansion, defined as an expansion index <0.8; malapposition refers to a physical gap between stent struts and the vessel wall. Malapposition triggers a cascade of disturbed flow, low shear stress, and endothelial dysfunction, in part through inhibition of the MER proto-oncogene tyrosine kinase receptor (MerTK), thereby promoting a prothrombotic and proinflammatory environment. Inadequate deployment is established as the primary mechanism for early thrombotic events, with underexpansion observed in 44.4% of stent thrombosis cases. ICI-guided PCI consistently demonstrates improved stent expansion and better clinical outcomes compared with angiography-alone strategies. Optical coherence tomography (OCT) offers superior resolution for detecting acute malapposition, whereas intravascular ultrasound (IVUS) provides more accurate vessel sizing in large or heavily calcified vessels. Consequently, a fundamental shift toward a “mechanism-based PCI strategy” is required, emphasizing that optimal stent sizing and deployment should be guided by ICI, rather than by angiographic endpoints alone. Wider adoption of imaging, specialized training in ICI for interventionalists, and the implementation of standardized definitions of malapposition are essential to minimize stent failure and achieve mechanistic excellence in coronary intervention.

## 1. Introduction

Stent undersizing and malapposition represent critical mechanical failures that continue to challenge contemporary percutaneous coronary intervention (PCI) despite technological advances in stent design, deployment techniques and current lesion preparation strategies. Both acute (AMal) and chronic stent malapposition (CMal) are associated with increased risk of early and late stent thrombosis (ST), future in-stent restenosis (ISR), higher rates of target lesion failure (TLF), and impaired long-term stent healing. Mechanical failures related to stenting architectures, including inadequate stent expansion (SE), stent malapposition (SM), and foreshortening, are often underestimated compared to patient and biological factors [1]. Notably, angiography often fails to detect clinically relevant SM, leading to systematic underestimation of its prevalence and clinical impact.

The prevalence of suboptimal stent deployment remains concerning, with studies demonstrating that SM occurs in 17.3% of longer lesions and stent undersizing (SU) in higher rates of MSA <5 mm^2^ in 9.6% of cases even with modern drug-eluting stents (DES) [2]. However, these figures likely underestimate the true burden of disease, as they are derived from studies employing intracoronary imaging (ICI); angiography-only cases may harbor substantially higher rates of undetected SM. These mechanical deficiencies create a substrate for both immediate and delayed complications, including acute ST, delayed healing, and late restenosis. Understanding the mechanisms underlying these complications requires comprehensive evaluation of deployment techniques, lesion characteristics, and the pathophysiological responses to inadequate vessel wall apposition.

The distinction between SU and SM is clinically important, though these conditions frequently coexist and share overlapping mechanisms. Stent underexpansion (SUE) refers to inadequate radial expansion of the stent in relation to the reference diameter of the distal vessel, typically defined as a stent expansion index less than 0.8 (always in accordance with each manufacturer’s specifications), while SM describes incomplete apposition of stent struts to the vessel wall, creating a gap between the strut and the arterial surface [3]. Both conditions frequently result from inadequate initial sizing decisions, compounded by insufficient lesion preparation, particularly when device selection relies on angiographic assessment alone-rather than ICI, that reveals true vessel dimensions.

The temporal evolution of malapposition is complex and clinically relevant, with both acute and chronic forms carrying significant prognostic implications. AMal present at implantation may persist indefinitely if not recognized and corrected, creating a sustained substrate for thrombosis and delayed healing. However, CMal may also develop over time in initially well-deployed stents due to thrombus resolution or positive vessel remodeling. When stents are implanted in vessels with significant thrombus burden, subsequent thrombus resolution can create gaps between previously apposed struts and the vessel wall. Similarly, positive remodeling in response to inflammatory stimuli or hypersensitivity reactions, can enlarge the vessel diameter, transforming initially well-apposed stents into malapposed configurations during the follow-up period.

Critically, both AMal and CMal are associated with increased risk of early and late ST, higher rates of TLF, and impaired long-term stent healing. The clinical consequences are independent of the timing of the malapposition development, as both forms expose thrombogenic surfaces to flowing blood, create regions of flow disturbance that delay endothelialization, and perpetuate inflammatory responses that impair normal healing. Angiography often fails to detect clinically relevant malapposition, as the two-dimensional projection cannot visualize the three-dimensional gap between struts and vessel wall unless the SM is severe and fortuitously aligned with the imaging plane. This detection failure leads to systematic underestimation of its prevalence and clinical impact, explaining why many “unexplained” late thrombotic events may actually result from unrecognized malapposition.

### 1.1 Intracoronary Imaging for Detection of Malapposition

ICI, particularly optical coherence tomography (OCT) and intravascular ultrasound (IVUS), is pivotal in identifying stent undersizing and malapposition, addressing fundamental limitations of angiographic assessment that contribute to systematic deployment defects. Angiography alone cannot reliably assess true vessel size in the setting of positive remodeling, characterize the depth and distribution of calcification, or distinguish vessel tapering from focal stenosis—all critical determinants of optimal device selection. Imaging-guided PCI has consistently demonstrated improved stent expansion, apposition, and clinical outcomes compared to angiography-alone strategies, establishing ICI as an essential tool for optimizing mechanical deployment and preventing the thrombotic and restenotic complications associated with undersizing and malapposition.

OCT offers superior spatial resolution for detecting AMal, with axial resolution approaching 10–15 micrometers, providing precise measurement of even small gaps between the stent struts and the vessel wall [4]. This exceptional resolution permits identification of subtle tissue that may prolapse between the struts, incomplete strut apposition, and stent edge dissections that could escape detection by other imaging modalities. OCT guidance led to greater stent expansion than standard of practice and also to lower rates of uncorrected dissection and malapposition than both IVUS and angiography guidance in randomized trials [5]. Pre-stenting decisions based on OCT, results in procedural change in more than 50% of cases in terms of stent length and diameter, while post-stenting OCT led to stent optimization in at least 27% of cases, with malapposition and underexpansion treated with post-dilatations [4].

In calcified coronary lesions where angiographic assessment is particularly unreliable, frequency-domain-optical coherence tomography (FD-OCT) has superior spatial resolution for detecting AMal enabled identification in all 12 patients compared to 10 of 12 patients with IVUS, and the extent of malapposition was significantly greater when assessed by OCT (20% versus 6%, *p* < 0.001) [6]. This enhanced detection capability is particularly valuable, as OCT can identify even small gaps between the struts and the vessel wall that IVUS may miss, due to its lower axial resolution. Conversely, IVUS provided accurate vessel sizing in these heavily calcified vessels, by penetrating beyond calcium deposits to visualize the external elastic lamina (EEL), enabling operators to confidently select appropriately sized devices despite extensive calcification. The complementary use of both modalities, IVUS for accurate vessel sizing and OCT for precise malapposition detection, represents an optimal strategy in complex calcified anatomy.

While IVUS provides accurate vessel sizing in larger or heavily calcified vessels, the penetration depth of IVUS (typically 4–8 mm compared to 1–2 mm for OCT) enables complete vessel wall visualization and assessment of EEL dimensions even in large proximal vessels where OCT may be limited by tissue attenuation. IVUS excels at characterizing calcium depth and determining true vessel size in vessels with extensive calcification, where accurate sizing is critical to prevent SU. Post-dilation increased the in-stent luminal area and reduced the extent of stent malapposition when assessed by FD-OCT, but changes were not statistically significant when measured by IVUS in calcified lesions, suggesting OCT’s superior sensitivity for detecting improvements in SM [6,7].

### 1.2 Mechanisms of Inadequate Stent Deployment

Stent undersizing commonly results from reliance on angiographic assessment alone, which fails to account for critical anatomical features that profoundly influence optimal device selection. In cases of stent undersizing, adequate sealing of the pre-existing atherosclerotic plaque is not achieved, resulting in persistent plaque exposure to the vessel lumen, despite the placement of metallic scaffold, potentially contributing to aggravation of the local endothelial shear stress (ESS) exerted on the arterial wall at the affected segment [8]. Vessel spasm, acute or chronic thrombus burden, and operator concern regarding edge dissection further bias practitioners toward conservative stent sizing, creating a systematic tendency toward undersized device selection. These limitations underscore why ICI has become increasingly critical for accurate vessel sizing and deployment optimization.

Coronary calcification represents one of the most significant predictors of SUE once a device has been selected, as rigid calcific deposits resist balloon expansion and prevent optimal stent apposition to the vessel wall. The extent of target lesion calcification as assessed by OCT is an important determinant of the expansion of second-generation DES, with mean stent expansion of 73.3 ± 8.7% for minimal stent diameter and 65.2 ± 12.0% for minimal stent area (MSA) [9]. SE defined by minimal stent diameter was significantly different among groups stratified by the arc and area of calcium, demonstrating the direct relationship between calcification burden and deployment adequacy. However, even when calcification is recognized, angiographic assessment alone cannot determine the depth, arc, and distribution of calcium necessary for planning optimal lesion preparation techniques.

Beyond calcification, several operator-dependent factors contribute to systematic undersizing, particularly in anatomically complex lesions. Vessel spasm, whether spontaneous or catheter-induced, can cause angiographic vessel narrowing that leads operators to select undersized devices based on the artificially reduced luminal diameter. Similarly, substantial thrombus burden obscures the true vessel size, biasing toward conservative device selection. Perhaps most perniciously, fear of edge dissection—a generally benign finding that rarely requires intervention—drives many operators toward deliberately undersized stents to avoid this radiographic finding. Indeed, the latter is often noted between interventionalists, even though undersized stents carry substantially higher risks of both thrombosis and restenosis than appropriately sized stents with minor edge dissections. Tapering anatomy presents additional challenges, as operators must decide whether to size according to the proximal or the distal vessel reference, often choosing conservatively to avoid potential complications.

### 1.3 Hemodynamic Consequences

The hemodynamic perturbations resulting from SU and SM create a milieu conducive to both thrombosis and restenosis through alterations in local flow patterns and endothelial shear stress (ESS) distribution. Hemodynamic shear stress, the frictional force exerted by blood flow on the endothelium, mediates vascular homeostasis, and disturbed flow patterns are most prevalent at sites of stent malapposition where recirculation zones develop [10]. Areas of low and oscillatory ESS promote endothelial dysfunction, inflammatory gene expression, and impaired mechanotransduction signaling. The presence of malapposed struts creates localized regions of flow separation and stasis, where platelet activation and thrombin generation can proceed unchecked by the normal antithrombotic properties of healthy endothelium, creating a sustained prothrombotic and pro-inflammatory environment that extends well beyond the immediate post-procedural period.

## 2. Pathophysiological Cascade Following Stent Malapposition

### 2.1 Disturbed Flow and Endothelial Dysfunction

Disturbed blood flow patterns resulting from SM trigger a cascade of endothelial dysfunction that fundamentally alters the vessel’s ability to resist thrombosis and inflammation. MerTK, a key receptor for efferocytosis expressed in endothelial cells, is sensitive to blood flow patterns and is inhibited by disturbed flow and oscillatory shear stress in primary human aortic endothelial cells [11]. MerTK deficiency aggravates atherosclerosis in disturbed flow areas through upregulation of endothelial dysfunction markers including IL-1β, NF-κB, TLR4, MAPK signaling, vWF, VCAM-1, and p22phox, while also inducing abnormal endothelial thickening accompanied by decreased endothelial efferocytosis. This mechanobiological response to flow disturbance represents a fundamental mechanism whereby deployment defects translate into pathological vessel responses.

Shear stress-induced endothelial cellular apoptosis occurs through interleukin-1 receptor-associated kinase 2-induced endoplasmic reticulum stress pathways. In patients with moderate-severe chronic obstructive pulmonary disease, oscillatory shear stress intervention induced significant decreases in brachial artery flow-mediated dilation and elevated circulating markers of endothelial cell apoptosis, effects that were abrogated by supplemental oxygen administration [12]. The RNA-seq data in human aortic endothelial cells incubated with apoptotic cells showed that disturbed flow promotes pro-inflammatory pathways and senescence pathways, creating a sustained environment hostile to vascular healing [11]. These molecular alterations impair the endothelium’s ability to perform its normal antithrombotic, anti-inflammatory, and barrier functions. The delayed re-endothelialization that characterizes malapposed stents perpetuates these disturbed flow effects, as exposed struts continue to generate abnormal hemodynamic patterns until complete cellular coverage is achieved.

A comprehensive understanding of the mechanisms underlying stent undersizing and malapposition may facilitate interruption of the pathological cascade initiated by disturbed coronary blood flow, ultimately reducing the risk of persistent stent malapposition and subsequent stent failure. Accordingly, meticulous procedural planning and optimal PCI execution remain essential for achieving durable procedural success.

ICI guidance plays a pivotal role in the prevention and correction of malapposition. Undetected gaps between stent struts and the vessel wall promote flow disturbances, delay endothelial healing, and perpetuate local inflammatory responses. Therefore, routine post-deployment assessment with IVUS or high-resolution OCT should be strongly considered to identify and correct suboptimal stent expansion and apposition. Beyond improving acute procedural outcomes, adequate stent optimization may also mitigate the risk of future ISR, as disturbed flow patterns and chronic vascular inflammation contribute to smooth muscle cell proliferation, neointimal hyperplasia, late ST, and TVF.

Prevention of microvascular dysfunction and the no-reflow phenomenon represents an additional therapeutic target. The judicious use of intracoronary vasodilators may help preserve microcirculatory integrity, particularly in complex interventions where abrupt restoration of coronary flow across sub-optimally apposed stent segments may exacerbate endothelial and microvascular injury.

Furthermore, advances in stent platform design may contribute to reducing the adverse biological consequences of malapposition. Thinner-strut and hemodynamically optimized stent architectures have been shown to minimize local flow separation and preserve physiological endothelial shear stress, thereby attenuating maladaptive inflammatory signaling pathways, including those associated with impaired MerTK-mediated efferocytosis. Such design improvements may promote vascular healing and reduce the long-term incidence of ISR, ST, and TVF.

### 2.2 Inflammatory Response

Localized inflammatory responses at sites of SM represent a critical component of the pathophysiological cascade leading to both thrombotic and restenotic complications. In patients with angiographic peri-stent contrast staining and/or incomplete stent apposition by IVUS, eosinophil fraction was significantly higher (10.6 ± 6.1% versus 5.8 ± 4.1%, *p* = 0.03), directly linking malapposition to inflammatory cell infiltration. These findings indicate that mechanical deployment defects create a nidus for chronic inflammation that persists long after the initial implantation.

The inflammatory milieu resulting from stent malapposition involves multiple cellular components and molecular mediators. Coronary stent implantation triggers inflammatory response, thrombogenic reactions, and smooth muscle cell hyperproliferation accompanied by delayed arterial healing and poor reendothelialization, leading to restenosis along with late stent thrombosis [13]. The rapid endothelialization of bare metal stents (BMS) is counterbalanced by inflammation-induced neointimal growth, while DES prevent leukocyte activation but impair endothelialization, delaying effective device integration into arterial walls [14]. CD31-mimetic stent coatings demonstrated that only these struts were fully endothelialized with no activated platelets or leukocytes at 7 days post-implantation, suggesting that modulating inflammatory responses can accelerate healing even in the presence of foreign material.

The temporal evolution of inflammation at sites of malapposition follows distinct patterns based on stent type and coating characteristics. At 28 days, neointima development over CD31-mimetic stents was significantly reduced compared to BMS, appearing as normal arterial media with absence of thrombosis contrary to DES [14]. Novel polymer-free everolimus-eluting stents fabricated using femtosecond laser showed reduced inflammation scores (1.9 ± 0.39) compared to conventional designs, demonstrating that surface characteristics profoundly influence the inflammatory response [15]. These data indicate that while mechanical factors initiate inflammation, the biochemical properties of stent surfaces modulate the inflammatory reaction’s magnitude and duration, with implications for long-term outcomes.

### 2.3 Platelet Activation and Coagulation

The exposure of thrombogenic surfaces at sites of SM creates conditions conducive to robust platelet activation and coagulation cascade initiation. Platelets can induce tissue factor expression in monocytes, a phenomenon inhibited by platelet P-selectin neutralization or integrin αIIbβ3 blocking [16]. This mechanism represents a rapid pathway by which activated platelets at malapposed stent struts can potentiate intracoronary coagulation through recruitment and activation of circulating monocytes. The formation of platelet-monocyte aggregates amplifies this procoagulant response, as the close cellular approximation facilitates efficient tissue factor-dependent thrombin generation.

### 2.4 Smooth Muscle Cell Proliferation

Neointimal hyperplasia resulting from smooth muscle cell proliferation and migration represents the final common pathway leading to in-stent restenosis, with mechanical deployment defects creating particularly fertile ground for this process. The uncontrolled proliferation and migration of vascular smooth muscle cells (VSMCs) is a critical step in the pathological process of restenosis caused by vascular intimal hyperplasia [17]. Platelet-derived growth factor-BB induces VSMC dedifferentiation, proliferation, and migration through activation of PI3K/AKT and p38 MAPK signaling pathways, processes that are amplified at sites of stent malapposition where platelet activation is enhanced.

**Fig. 1.**
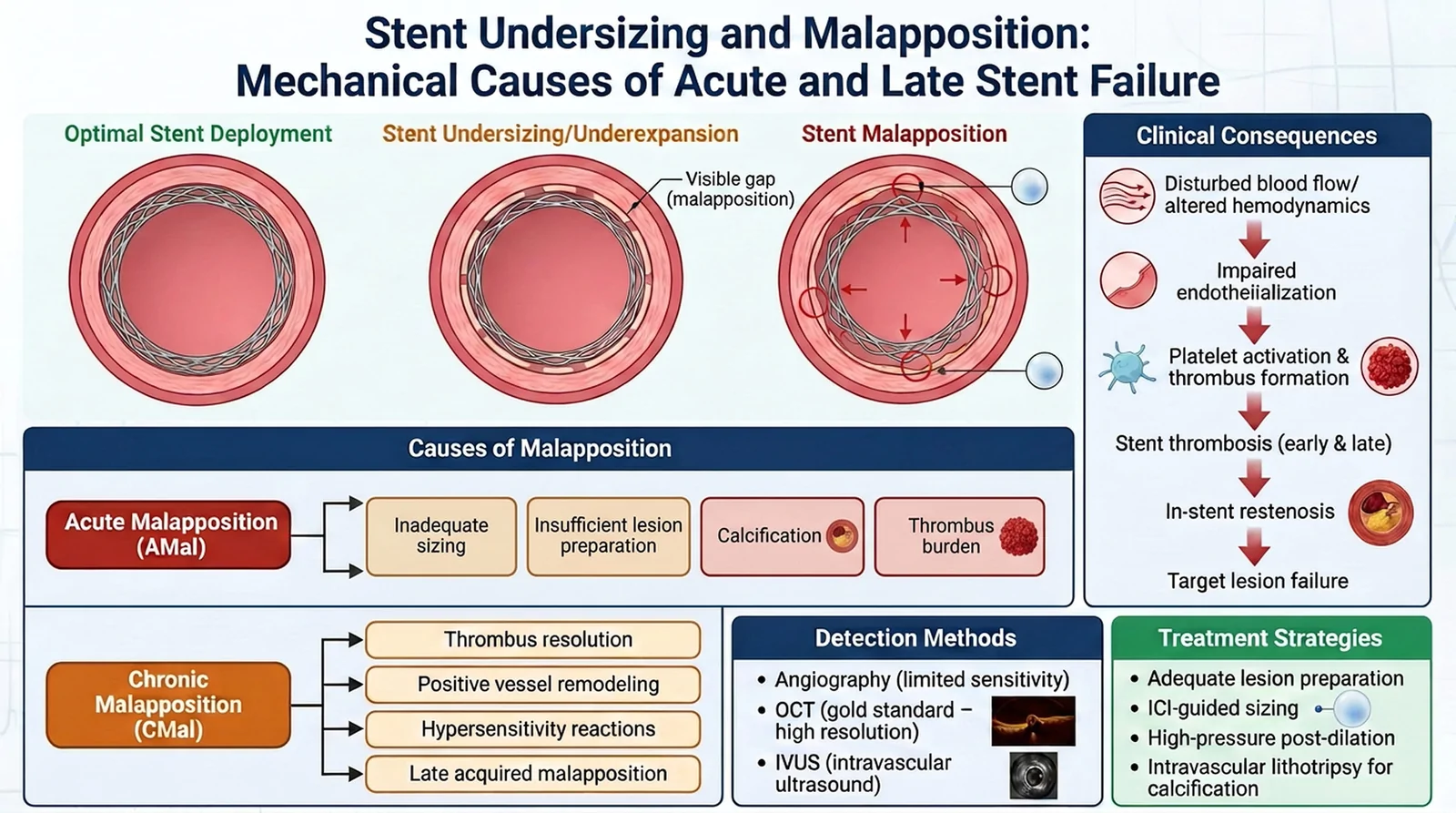
**Mechanisms of acute and chronic stent malapposition - schematic illustration**. OCT, Optical coherence tomography; ICI, intracoronary imaging.

Mechanisms linking stent undersizing and malapposition to acute and late stent failure are illustrated in Fig. 1.

## 3. Clinical Outcomes and Risk Stratification

### 3.1 Acute and Subacute Stent Thrombosis

Acute and subacute ST represent the most catastrophic early complications of inadequate stent deployment, with distinct temporal patterns and underlying mechanisms. Both AMal and CMal are associated with increased risk of early and late ST, creating a persistent substrate for catastrophic events that extends well beyond the immediate post-procedural period. In a prospective multicenter OCT study of 217 patients presenting with stent thrombosis, the predicted average probability that any frame had uncovered struts was 99.3% for acute ST, 96.6% for subacute ST, 34.3% for late ST, and 9.6% for very late ST [3]. Malapposed struts showed a similar temporal decline, present in 21.8% of frames in acute ST, 8.5% in subacute ST, 6.7% in late ST, and 2.0% in very late ST [3].

The most common dominant finding adjudicated for acute ST was uncovered struts (66.7% of cases), while for subacute ST, uncovered struts (61.7%) and SUE (25.5%) predominated. These data definitively establish inadequate deployment as the primary mechanism of early thrombotic events. Critically, these sites of SM were invisible on angiography, leading to underestimation of their prevalence and contribution to thrombotic events. Many cases attributed to “unexplained” ST likely resulted from angiographically silent SM that could have been detected and corrected with routine use of ICI.

SUE, defined as stent expansion index less than 0.8, was observed in 44.4% of patients presenting with ST across all time periods [3]. The reference vessel diameter was relatively modest at 2.9 ± 0.6 mm with mean reference vessel area of 6.8 ± 2.6 mm^2^, suggesting that device selection may have been inappropriate for vessel size or that achieved expansion was inadequate despite appropriate sizing. The high prevalence of underexpansion in thrombotic events underscores the critical importance of achieving optimal stent dimensions, as even modest degrees of underexpansion create hemodynamic disturbances sufficient to trigger thrombosis. Risk factors for early ST include procedural factors such as malapposition and underexpansion, number of implanted stents, length, persistent slow coronary blood flow, and dissections, especially flow-limiting ones [18].

The procedural success rate of IVL for treating SUE reached 72.6%, with only one cardiac death (1.6%) during 30-day follow-up and no target lesion revascularization or target vessel myocardial infarction, demonstrating that correction of mechanical defects can salvage even severely underexpanded stents [19]. This finding is particularly relevant for cases where initial conservative sizing due to vessel spasm or thrombus burden resulted in undersized device selection, as subsequent optimization with intravascular lithotripsy (IVL) can achieve adequate expansion once these transient factors resolve.

### 3.2 Late and Very Late Complications

The mechanisms underlying late and very late ST differ fundamentally from early events, though both AMal and CMal remain associated with increased risk throughout all time periods. Neoatherosclerosis emerges as a dominant pathological process in delayed failures, but CMal developing from thrombus resolution or vessel remodeling continues to contribute substantially to late events. In patients presenting with very late ST, uncovered stent struts were a common dominant finding in DES, while neoatherosclerosis was a common dominant finding in BMS [3]. For late ST, the most common dominant findings were uncovered struts (33.3%) and severe restenosis (19.1%), whereas for very late ST, neoatherosclerosis (31.3%) and uncovered struts (20.2%) predominated.

This temporal evolution reflects the transition from deployment-related mechanisms to progressive atherosclerotic disease within the stented segment, though incomplete strut coverage and malapposition remain relevant even at extended time points. The persistence of SM as a contributing factor to very late events underscores that impaired long-term stent healing represents a fundamental consequence of inadequate initial deployment. Angiography systematically fails to detect these sites of late malapposition, which may be attributed to “late stent failure” without recognition of the underlying mechanical substrate that could have been identified and addressed within ICI.

Localized hypersensitivity reaction, delayed arterial healing, and neoatherosclerosis inside the stent have been suggested as underlying pathologic mechanisms of very late stent thrombosis of DES [20]. Evidence for fragments of atherosclerotic plaques in aspirated thrombi were observed in only 13% of patients with DES very late ST, suggesting that while neoatherosclerosis contributes to late events, other mechanisms including persistent inflammation and inadequate healing remain important.

The issue of late in-stent restenosis, including neoatherosclerosis after DES implantation, especially in high-risk patients and complex lesions, still exists as a challenge to be overcome [21] and will be covered in the following paragraph in detail.

### 3.3 In-Stent Restenosis

The distinction between neointimal hyperplasia and neoatherosclerosis has prognostic implications, as hyperplasia typically occurs earlier and may respond to repeat revascularization, while the latter develops over the years and creates a vulnerable substrate prone to plaque rupture and thrombosis similar to native coronary artery disease. In-stent restenosis (ISR) represents the most common manifestation of stent failure, occurring through mechanisms intimately linked to initial deployment adequacy and subsequent healing responses. Neoatherosclerosis represents an increasingly recognized mechanism of late restenosis, particularly in DES, where delayed healing creates a substrate for atherosclerotic plaque development within the neointima. Unravelling the key molecular and signal pathways involved in ISR is crucial to identify appropriate therapeutic strategies aimed at abolishing the Achilles heel of PCI [22].

Both AMal and CMal are associated with higher rates of TLF, which encompasses restenosis as a major component alongside thrombosis and target vessel myocardial infarction. The initial drawbacks of first-generation DES, such as delayed endothelial healing and subsequent risk of late stent thrombosis, have been improved upon by current generation (3rd) DES, yet late events persist in contemporary practice. The incidence of ISR has reduced from 30% at 6 months with BMS to 7% at 4 years with DES, yet its occurrence remains relevant in absolute terms because of the dimensions of the population treated with PCI [22]. Risk factors associated with ISR are usually divided into patient-related, genetic, anatomic, stent, lesion, and procedural characteristics. However, inadequate stent deployment—particularly malapposition—represents an underrecognized but critical procedural risk factor that angiography systematically fails to detect, leading to underestimation of its contribution to restenotic outcomes.

The relationship between stent underexpansion and restenosis is well-established but mechanistically complex. ICI-guided PCI has consistently demonstrated improved stent expansion, apposition, and clinical outcomes, addressing the fundamental limitations of angiographic assessment that lead to systematic undersizing. Smaller MSA emerged as an independent predictor of 2-year target lesion failure in the ILUMIEN IV, involving 2487 patients with diabetes mellitus or high-risk lesions. The trial demonstrated that OCT-guided PCI achieved superior procedural metrics including greater stent expansion, better apposition, and more frequent optimization compared to angiography-guided procedures [23]. Suboptimal deployment decreased from 30.1% to 7.6% after post-dilatation when IVUS imaging was employed, with residual plaque burden reduced to 4.5–5.7% and minimal stent area optimized to 6.3–6.5 mm^2^ [2]. These procedural improvements translate into clinical benefits, as smaller MSA—the metric most accurately assessed by imaging—independently predicted 2-year TLF with a hazard ratio of 0.76 per 1 mm^2^ increase (95% CI 0.68–0.89, *p* < 0.0001) [23].

Additionally, smaller intra-stent flow area independently predicted ischemia-driven target lesion revascularization (ID-TLR), while proximal edge dissection was associated with both target lesion failure and ID-TLR. These findings establish that achieving adequate stent dimensions is not merely a procedural endpoint but a critical determinant of long-term clinical success.

For ISR treatment, using dedicated devices, like the super non-compliant OPN™ balloon at very high pressures, is generally safe. As suggested by current published data, their use might lead to a low rate of TLF during long-term follow-up, but further randomized controlled trials are warranted [24].

### 3.4 Clinical Significance of Angiographically Silent Malapposition

The clinical consequences of angiographically silent malapposition are substantial. Malapposed struts present in 21.8% of frames in acute ST and persist in 2.0% of frames even in very late ST were invisible on the angiograms that deemed these deployments acceptable [3]. These undetected mechanical substrates create persistent regions of disturbed flow, delayed endothelialization, and enhanced thrombogenicity that translate directly into adverse clinical events.

The association between malapposition and impaired long-term stent healing extends well beyond the immediate post-procedural period, as incompletely apposed struts may never achieve complete endothelial coverage, maintaining a perpetual thrombotic risk. This explains why some patients experience “unexplained” late thrombotic events despite apparent procedural success—the underlying mechanical substrate was present from implantation but invisible to angiographic assessment, and CMal developing from thrombus resolution or vessel remodeling similarly escapes angiographic detection during follow-up.

### 3.5 Predictive Imaging Parameters

Quantitative parameters derived from ICI provide powerful tools for predicting adverse events and guiding procedural optimization, addressing the fundamental detection limitations of angiography. The most important OCT-derived post-DES predictors of both safety and effectiveness outcomes were parameters related to stent area, expansion and flow, proximal edge dissection, and stent length [23]. MSA emerged as the single most predictive metric, with each 1 mm^2^ increase in MSA associated with a 24% reduction in TLF risk. Smaller minimal SE was the independent predictor of ST, establishing a quantitative threshold below which thrombotic risk substantially increases.

Stent expansion index, typically calculated as the ratio of MSA to average reference lumen area, provides a normalized metric accounting for vessel size variability. OCT-guided rotational atherectomy achieved superior percent SE (83 ± 15%) compared to IVUS-guided procedures (72 ± 16%, *p* = 0.0004), with this difference translating into improved procedural outcomes [7]. Although target lesion revascularization at 1 year was lower in the OCT-guided group (6.8% versus 11.6%), this difference did not reach statistical significance, possibly reflecting inadequate sample size to detect clinically meaningful differences in hard endpoints.

Edge dissections represent another critical predictor identified through high-resolution imaging, with proximal edge dissections independently predicting both TLF and ID-TLR [23]. Post-dilation increased the in-stent luminal area and reduced the extent of SM when assessed by FD-OCT, demonstrating that procedural optimization guided by imaging can correct deployment defects before their clinical consequences manifest [6].

## 4. Prevention-Treating Strategies and Future Directions

### 4.1 Management Strategies for Preventing Stent Undersizing and Malapposition

#### Improving Aspects of Appropriate Vessel Lumen Sizing and Adequate Lesion Preparation

Effective management of SU and SM requires a systematic approach that addresses each phase of the procedure, from initial assessment through post-deployment optimization and surveillance. The cornerstone of prevention is appropriate vessel sizing using ICI prior to stent selection, which overcomes the fundamental limitations of angiographic assessment that lead to systematic undersizing in vessels with diffuse disease, positive remodeling, heavy calcification, or tapering anatomy. Imaging-guided vessel sizing enables operators to confidently select appropriately sized devices without the conservative biases driven by vessel spasm, thrombus burden, or fear of edge dissection.

Another key concept is the effective preparation of the targeted lesions, utilizing any necessary method, or combined treatment whenever needed. In resistant lesions with heavy calcification, lesion preparation with calcium-modifying techniques is essential to achieve adequate stent expansion. Numerous trials have underscored SUE, as the most important factor of future stent failure. Consequently, contemporary PCI cannot be based on conventional balloon angioplasty, prior to stenting. Modern techniques, including cutting or scoring balloons, rotational (RA)-or orbital atherectomy (OA), Excimer Laser Coronary Angioplasty (ELCA) in cath labs familiar with this method, and intravascular lithotripsy (IVL) are becoming part of everyday practice [25].

The choice of balloon type and inflation pressure significantly impacts not only the mandatory step of adequate lesion preparation prior to stenting, but also the final stent dimensions, once a device has been implanted. Predilatation using non-compliant balloons (NCBs) at high pressures (24 ± 7 atmospheres) resulted in better SE compared to semi-compliant balloons (SCBs) (14 ± 3 atmospheres), with SE index improving from 0.85 ± 0.14 to 0.94 ± 0.13 (*p* = 0.02) after post-dilation with NCBs [26]. The mechanism whereby high-pressure inflation with NCBs improves expansion, likely involves more effective fracture and displacement of calcific deposits, allowing greater radial force transmission to the vessel wall, therefore resulting in more luminal gain. Using SCBs only for predilatation might lead to inadequate SE, and post-dilatation with NCBs might only partially correct this deficiency.

Cutting or scoring balloons represent a well-established calcium-modifying technique, creating controlled disruptions in calcific plaque that facilitate subsequent balloon expansion without extensive ablation. These devices are particularly useful for moderately calcified lesions where full atherectomy may be unnecessary, but standard balloon angioplasty proves inadequate.

Atherectomy is the standard strategy when treating balloon uncrossable lesions. RA, OA or even ELCA, combined with imaging guidance, optimizes lesion preparation in severely calcified substrates, enabling more predictable outcomes. The aforementioned applications can also become the initial part of a more sophisticated preparation pathway, involving also IVL as sequential step (e.g., emerging cases of RA + IVL, called RotaTripsy etc.) [27].

Finally, re-intervention in selected cases of clinically relevant late malapposition associated with adverse events may be necessary to address CMal developing from thrombus resolution or positive vessel remodeling.

### 4.2 Management Strategies for Treating Stent Undersizing, Underexpansion and Malapposition

High-pressure post-dilatation during the index procedure with NCBs sized to the reference vessel represents a critical component, correcting residual underexpansion and malapposition that may persist even after initial deployment. ICI is recommended to further improve final outcomes, whenever feasible by logistics. However, differences between centers have been noted. Large prospective data from Netherlands, suggests that post‐dilatation is more often used in cases of complex interventions, and when ICI or calcium modification techniques are followed [28].

To deal with suboptimal stent expansion, a lot of different commercially available devices-on top of conventional balloon utilization, have been assigned to our arsenal. Prior stent dimensions, clinical factors, SUE severity, vessel calcification, details derived from angiography and ICI, and frailty play a cardinal role in decision-making when tackling this entity [22].

IVL represents a transformative technology for managing severely calcified lesions prone to underexpansion, utilizing sonic pressure waves to fracture calcium deposits and facilitate stent expansion. Therefore, it has emerged as a powerful adjunctive technique for treating stent underexpansion, achieving procedural success (relative SE >80%) in 72.6% of patients with established underexpansion [19]. Both SUE and stenotic area improved significantly following IVL application, from 58.5% to 11.4% and from 82.6% to 21.5%, respectively (both *p* < 0.001). IVL. ICI confirmed increased SE following IVL from 37.5% to 86.0% (*p* < 0.001) by OCT and from 57.0% to 89.0% (*p* = 0.002) by IVUS, demonstrating effectiveness across different imaging modalities. The safety profile was excellent, with device-oriented composite endpoint occurring in only 1.6% of patients at 30 days.

Lately, new combination therapies have been tested in some cases, involving the combined effect of ELCA and super high-pressure balloons to treat single or multiple layers of stent with severe under-expansion [29].

A holistic approach relating several technical factors to the use of ICI in contemporary PCI, either beforeor after stent placement is given in Table 1 (Ref. [2,4,5,6,7]).

**Table 1.** **Comparative strengths of OCT and IVUS for identifying stent deployment defects**.

Parameter	OCT	IVUS	Clinical application
Axial resolution	10–15 μm (Superior)	100–150 μm	OCT detects subtle malapposition invisible to IVUS
Penetration depth	1–2 mm	4–8 mm (Superior)	IVUS visualizes complete vessel wall in large vessels
Malapposition detection	Pivotal—detects gaps as small as 40 μm	Detects larger gaps (>100 μm)	OCT superior for acute malapposition detection [6]
Extent of malapposition	20% detected [6]	6% detected [6]	OCT detects 3-fold greater extent (*p* < 0.001)
Vessel sizing	Limited in large vessels	Pivotal—accurate EEL measurement	IVUS superior for large/calcified vessels [4]
Calcium assessment	Arc and thickness (superficial)	Depth and distribution	OCT for superficial, IVUS for deep calcium
Strut-level analysis	Excellent—individual strut apposition	Limited	OCT for detailed strut coverage assessment
Post-dilation guidance	Optimal—detects improvement in malapposition	Less sensitive to changes [6]	OCT for verifying malapposition correction
Stent expansion measurement	Precise MSA, asymmetry	Reliable MSA, EEL-based expansion	Both effective; OCT more precise [7]
Optimization rate	Changes in 27%+ of cases [4]	Reduces suboptimal deployment 30% → 7.6% [2]	Both improve deployment vs. angiography
Procedural changes	>50% change in stent sizing [4]	Enables confident sizing in difficult anatomy	Both overcome angiographic limitations
Clinical outcomes	Improved expansion, ↓ malapposition [5]	Superior to angiography [4]	Imaging-guided PCI consistently demonstrates improved expansion, apposition, and outcomes
Optimal use	• Detecting acute malapposition • Strut assessment • Post-dilation verification • Small-medium vessels	• Vessel sizing (large vessels) • Heavy calcification • Calcium depth • EEL visualization	Complementary; selection based on scenario

Note: Both OCT and IVUS are pivotal in identifying stent undersizing and malapposition. OCT offers superior spatial resolution for detecting acute malapposition; IVUS provides accurate vessel sizing in larger or heavily calcified vessels. Imaging-guided PCI has consistently demonstrated improved stent expansion, apposition, and clinical outcomes. EEL, external elastic lamina; MSA, minimal stent area; PCI, percutaneous coronary intervention.

#### Stent Design Innovations

Contemporary stent design optimization focuses on multiple interrelated parameters including strut thickness, material properties, drug and polymer characteristics, and geometric architecture. The design of stent platforms is constantly improving to maximize efficacy and safety, with development including adoption of new materials for scaffold production, new design types, improved overexpansion abilities, new polymers coating, and improved antiproliferative agents [30]. Strut thickness reduction has emerged as a particularly important design modification, as thinner struts generate less flow disturbance, require less neointimal coverage to achieve a smooth luminal surface, and may facilitate re-endothelialization. However, strut thickness reduction must be balanced against the need for adequate radial strength to resist elastic recoil and maintain vessel patency, particularly in calcified lesions.

Self-expanding stents (SES) with auxetic metamaterial properties represent an innovative approach to addressing deployment challenges, particularly regarding malapposition and longitudinal deformation. Auxetic materials with negative Poisson’s ratio can enhance mechanical properties of structures, including fracture toughness, indentation and shear resistance, and vibration energy absorption, which will help resolve drawbacks due to mechanical failures [1]. The topologically optimized auxetic architecture provides a new solution for developing novel stenting structures, that can overcome potential limitations of conventional stents associated with mechanical structures, while maintaining their valuable features. However, SES have not achieved widespread adoption in coronary interventions due to concerns regarding deliverability, placement accuracy, and precise matching of stent size and shape to the vessel.

Novel coating strategies aim to accelerate re-endothelialization while minimizing inflammation and smooth muscle cell proliferation. An endothelium-mimicking coating capable of rapid regeneration of a competently functioning new endothelial layer achieved stable nitric oxide generating rates at physiological levels combined with high heparin conjugation efficacy [13]. Apart from enhanced anti-thrombogenicity and anti-inflammation, this coating promoted re-endothelialization by regulating relevant gene expressions, hence preventing restenosis *in vivo*. CD31-mimetic coating demonstrated that only these struts were fully endothelialized with no activated platelets/leukocytes at 7 days post-implantation, with neointima development significantly reduced at 28 days appearing as normal arterial media [14]. These advances suggest that surface engineering strategies may partially compensate for mechanical deployment defects by creating more biocompatible interfaces.

### 4.3 Personalized Approach and Future Research

#### 4.3.1 Toward a Mechanism-Based PCI Strategy: The Imperative for Change

A fundamental shift toward a mechanism-based PCI strategy is required, emphasizing optimal stent sizing and deployment rather than angiographic endpoints alone. The accumulated evidence demonstrating that both AMal and CMal are associated with increased risk of early and late ST, higher rates of TLF, and impaired long-term stent healing—coupled with the systematic failure of angiography to detect these critical deployment defects—mandates a paradigm change in how procedural success is defined and evaluated. Lumen size plays a pivotal role in predicting possible adverse effects, with potential worse prognosis for vessels of smaller caliber in comparison to larger ones. For instance, in a trial by Wu X et al. [31], despite AMal in the left main coronary artery was not uncommon post-PCI, it did not correlate with adverse cardiac events in the follow-up period.

Angiographic assessment, while providing useful anatomical information about stenosis severity and flow, is fundamentally inadequate for ensuring optimal mechanical deployment. The evolution from angiographic endpoints to mechanism-based PCI represents a fundamental reconceptualization of procedural success. ICI is pivotal in identifying stent undersizing and malapposition—mechanical defects that angiography systematically fails to detect but that profoundly impact clinical outcomes. Imaging-guided PCI has consistently demonstrated improved stent expansion, apposition, and clinical outcomes, establishing the evidence base for a new standard of care. As shown by recent data, in real-world clinical practice, the rates of both OCT- and IVUS-guided PCI have risen. It is interesting that ICI-guided PCI has also been associated with a lower risk of myocardial infarction recurrence, compared with angiography-guided PCI in patients with a prior ischemic event [32].

The path forward requires coordinated action across multiple domains: wider adoption of ICI through addressing cost, workflow, and reimbursement barriers; comprehensive operator education emphasizing mechanism-based optimization principles; development and implementation of standardized malapposition definitions enabling consistent research and quality improvement; focused investigation defining clinically relevant malapposition thresholds and optimal correction strategies; and continued innovation in imaging technology, computational modeling, and stent design. As these initiatives advance, the field will move progressively toward a future where stent failure becomes increasingly rare—not because we have developed more forgiving devices, but because we have achieved mechanistic excellence in deployment that eliminates the substrates of thrombosis, restenosis, and impaired healing from the outset. A thorough risk assessment is required due to the complex etiology of stent thrombosis and in-stent restenosis, with the most effective strategy being prevention through identification of patient-, lesion-, device-, and procedure-related predictors [33].

#### 4.3.2 Implementation Strategies: Wider Adoption, Education, and Standardization

Wider adoption of ICI represents the cornerstone of transitioning to mechanism-based PCI, yet multiple barriers impede universal implementation. Cost considerations, procedural time constraints, reimbursement limitations, and the learning curve associated with image acquisition and interpretation all contribute to suboptimal imaging utilization rates in contemporary practice. However, the clinical and economic consequences of stent failure—including catastrophic events like stent thrombosis that carry 20–40% mortality rates, repeat revascularization procedures, and prolonged dual antiplatelet therapy with associated bleeding risks—argue compellingly for investment in imaging technology and training. As imaging platforms become more accessible, user-friendly, and efficient, and as evidence continues to demonstrate clinical benefits, the cost-benefit equation increasingly favors routine imaging use, particularly in complex lesions and high-risk patients.

Operator education is essential to maximize the benefits of imaging-guided PCI. Understanding imaging artifacts, recognizing normal versus abnormal deployment features, interpreting quantitative measurements, and translating imaging findings into procedural decisions require dedicated training beyond basic interventional skills. Educational initiatives should put emphasis on:

(1) appropriate vessel sizing using ICI prior to stent selection, overcoming biases toward conservative sizing;

(2) recognition of malapposition patterns and their clinical significance;

(3) high-pressure post-dilatation with non-compliant balloons sized to the reference vessel as routine practice rather than reserved for suboptimal cases;

(4) lesion preparation with calcium-modifying techniques guided by imaging assessment of plaque morphology; and

(5) re-intervention strategies for clinically relevant late malapposition. Simulation-based training, case-based learning, and mentorship programs can accelerate the learning curve and promote standardized approaches to imaging interpretation and procedural optimization.

Standardized definitions of malapposition are critically needed to enable consistent research, quality improvement initiatives, and clinical decision-making. Current literature employs variable definitions—some based on absolute gap distance (>200 μm, >400 μm), others on strut-to-wall distance relative to strut thickness, and still others on qualitative assessment of incomplete apposition. This heterogeneity precludes meaningful comparison across studies and impedes development of evidence-based thresholds for intervention. Proposed standardized criteria should address:

(1) minimum gap distance constituting malapposition (accounting for measurement precision and biological variability);

(2) minimum longitudinal extent requiring intervention (focal vs. diffuse malapposition); (3) minimum circumferential involvement (arc of malapposition);

(4) volumetric metrics integrating gap distance, longitudinal extent, and circumferential involvement; and

(5) temporal classification distinguishing acute, persistent, and LASM.

Consensus definitions endorsed by major cardiovascular societies would facilitate multicenter research, enable quality benchmarking, and guide clinical practice.

#### 4.3.3 Future Research Priorities: Defining Thresholds and Optimizing Strategies

Future research should focus on defining thresholds of clinically relevant malapposition that predict adverse events and warrant intervention. While it is established that both acute and chronic malapposition are associated with increased thrombosis risk, higher target lesion failure rates, and impaired healing, the quantitative relationship between malapposition severity and event rates remains incompletely characterized.

Large-scale prospective registries employing standardized malapposition definitions and systematic post-procedural imaging are needed to determine:

(1) the minimum gap distance that significantly increases thrombotic risk;

(2) whether longitudinal extent or circumferential involvement better predicts events;

(3) how malapposition location (ostial, mid-segment, bifurcation) influences clinical significance;

(4) whether LASM carries different risk than persistent acute malapposition; and

(5) how patient and lesion characteristics modify malapposition-associated risk.

Such data would enable development of risk stratification algorithms identifying which sites of malapposition require aggressive intervention versus conservative management.

Similarly, optimal correction strategies for identified malapposition require systematic investigation. While high-pressure post-dilatation with NCBs represents the first-line approach, evidence-based guidance on balloon sizing (reference vessel vs. larger), inflation pressure (standard vs. very high pressure), inflation duration, and the role of adjunctive technologies (intravascular lithotripsy, cutting balloons) in resistant cases is lacking.

For LASM from positive vessel remodeling, comparative effectiveness research evaluating balloon dilation alone, additional stent implantation, and conservative management with intensified antiplatelet therapy is needed. The optimal timing of re-intervention—immediate upon detection versus surveillance with intervention only upon clinical events—represents another critical knowledge gap.

Randomized trials comparing imaging-guided optimization strategies versus angiography-alone approaches in specific high-risk populations (acute coronary syndromes with large thrombus burden, heavily calcified lesions, bifurcations, long lesions) would provide definitive evidence for imaging’s clinical value. The decision to intervene on late malapposition must balance procedural risks against the natural history of untreated malapposition.

Malapposition in patients with very late stent thrombosis was associated with significantly higher eosinophil fractions in aspirated thrombi (10.6 ± 6.1% versus 5.8 ± 4.1%, *p* = 0.03), suggesting ongoing hypersensitivity reactions at sites of incomplete apposition [20]. When late malapposition is discovered incidentally during surveillance imaging in asymptomatic patients, conservative management with intensified antiplatelet therapy may be appropriate. However, symptomatic patients, those with very large malapposition areas, or patients presenting with acute events attributable to malapposition warrant consideration for mechanical intervention.

Emerging technologies offer promise for further advancing mechanism-based PCI. Artificial intelligence and machine learning algorithms applied to imaging data may enable automated detection and quantification of malapposition, reducing operator dependence and enabling real-time decision support. Integration of computational fluid dynamics with patient-specific imaging data could predict regions at risk for disturbed flow and identify deployment defects most likely to generate clinically significant hemodynamic disturbances. Novel stent designs incorporating sensors that detect incomplete apposition and trigger alerts could provide immediate feedback during deployment. Bioresorbable scaffolds that eliminate the permanent mechanical substrate of malapposition represent a conceptually attractive approach, though clinical experience has revealed that these devices are even more sensitive to deployment adequacy than metallic stents, reinforcing rather than obviating the need for meticulous imaging-guided optimization.

## 5. Conclusion

Both acute and chronic malapposition are associated with increased risk of potential stent failure, manifested as early and late stent thrombosis, future stent restenosis, higher rates of target lesion failure, and impaired long-term stent healing.

Several procedural and lesion-related risk factors may lead to this complication, highlighting the limitations of classic angiography in detecting this entity. These further underscore the importance of intracoronary imaging in identifying stent undersizing and malapposition, and eventually in guiding the subsequent PCI. Use of ICI in both diagnosis and treatment, has led to improved stent expansion, apposition, and clinical outcomes in plenty of RCTs and meta-analyses.

In conclusion, a shift towards a mechanism-based PCI strategy, aiming for optimal stent sizing and deployment-rather than acting on traditional angiographic endpoints alone, is required. Such a philosophy should be widely adopted by interventionalists worldwide.

This imperative shift in practice can be dramatically aided by a number of actions, like the implementation of dedicated training in intracoronary imaging, questioning our perspective regarding older habits like angiography-only decisions, and the need for an international adoption of a standardized definition regarding malapposition. Design of large, randomized trials, confronting these matters, is of paramount importance.
